# Factors associated with health-related quality of life in patients with functional dyspepsia

**DOI:** 10.1186/s12955-018-0913-z

**Published:** 2018-05-02

**Authors:** Ibnu Fajariyadi Hantoro, Ari Fahrial Syam, Endang Mudjaddid, Siti Setiati, Murdani Abdullah

**Affiliations:** 1 Departement of Internal Medicine, Faculty of Medicine Universitas Indonesia, Cipto Mangunkusumo Hospital, Jalan Diponegoro No. 71, Jakarta, 10430 Indonesia; 2 Division of Gastroenterology, Departement of Internal Medicine, Faculty of Medicine Universitas Indonesia, Cipto Mangunkusumo Hospital, Jalan Diponegoro No. 71, Jakarta, 10430 Indonesia; 3 Division of Psychosomatic, Departement of Internal Medicine, Faculty of Medicine Universitas Indonesia, Cipto Mangunkusumo Hospital, Jalan Diponegoro No. 71, Jakarta, 10430 Indonesia; 4 Clinical Epidemiology Unit, Departement of Internal Medicine, Faculty of Medicine Universitas Indonesia, Cipto Mangunkusumo Hospital, Jalan Diponegoro No. 71, Jakarta, 10430 Indonesia

**Keywords:** Functional dyspepsia, Health related quality of life

## Abstract

**Background:**

Health-related quality of life (HRQoL) assessment is important for patients with functional dyspepsia. However, no studies have assessed factors associated with HRQoL reduction in such patients in an Asian population. This study aimed to determine the contribution of clinical, psychosocial, and demographic factors to HRQoL in affected patients in Indonesia.

**Methods:**

In a cross-sectional study, we recruited 124 patients in a tertiary hospital with functional dyspepsia according to Rome III criteria. HRQoL was measured using the Medical Outcomes Study Short-Form 36 (SF-36) physical component summary (PCS) and mental component summary (MCS) and compared with 2009 United States population norms. The factors investigated were age, gender, symptom severity, education level, employment status, anxiety, depression, and ethnicity. Factors associated with reduced HRQoL were identified using linear regression analysis.

**Results:**

All domains of HRQoL except vitality were impaired in patients with functional dyspepsia. The mean PCS was 42.3 (SD = 8.4); and the mean MCS was 47.8 (SD = 10). Increasing age (*p* = 0.002), female gender (*p* = 0.006), low-to-mid education level (*p* = 0.015) and greater symptom severity (*p* < 0.001) were significantly associated with impaired PCS (R^2^ = 0.36). Female gender (*p* = 0.047), greater symptom severity (*p* = 0.002), anxiety (*p* = 0.001), and depression (*p* = 0.002 were all significantly associated with an impaired MCS (R^2^ = 0.41). There were no significant associations between HRQoL and with ethnic group (Javanese/non-Javanese) or employment status.

**Conclusions:**

There was significant HRQoL impairment in these patients with functional dyspepsia in Indonesia. Anxiety, depression, increasing age, female gender, greater symptom severity, and low-to-mid education level were significant factors associated with low HRQoL.

**Trial registration:**

ClinicalTrials.gov NCT03321383. Registered 18 October 2017 retrospectively registered.

## Background

Functional dyspepsia is defined as the presence of symptoms thought to originate in the gastroduodenal region, in the absence of any organic, systemic, or metabolic disease that is likely to explain the symptoms [1]. Dyspepsia symptoms are commonly seen in the community and in clinical practice [2]. The prevalence of functional dyspepsia in Asian populations is 7–20% [3]. Several Asian hospitals have also reported that 50–80% of patients with dyspepsia are found to have functional dyspepsia [2, 4].

HRQOL describes health status based on patients’ perceptions. It consists of physical, psychological, and social function domains [5, 6]. Functional dyspepsia is not associated with increased mortality so HRQoL assessment is important to understand the impact of disease and treatments on patients [7–9]. Previous studies have demonstrated impaired HRQoL in patients with functional dyspepsia [10–16]. Inside factors such as the type of illness, the nature and severity of symptoms, any comorbidities, health knowledge, anxiety, and depression can influence HRQoL. It is also affected by outside factors such as socioeconomic status, social support, culture, demographic and medical care quality and access [17]. Studies in patients with functional dyspepsia have found the contribution of clinical factors such as symptom severity, psychosocial factors such as anxiety, and depression, and demographic factors such as age and gender in decreasing the patients’ HRQoL [10, 12–14]. However, studies on factors associated with HRQoL in patients with functional dyspepsia are rare and have been limited to Western populations [10, 12–14], eventhough there are differences in clinical and epidemiological characteristics between Asian and Western patients [18]. Previous studies have also not evaluated the relationship between education level and ethnicity on the quality of life of patients with functional dyspepsia.

The objectives of this study were to assess the impact of functional dyspepsia on HRQoL among patients in a tertiary hospital in Indonesia. We also aimed to determine the contribution of clinical, psychosocial, and demographic factors to the HRQoL of such patients.

## Methods

### Study populations

A cross-sectional study was carried out at the Gastroenterology Clinic Cipto Mangunkusumo Hospital between August 2016 and June 2017. A total of 124 consecutive patients aged 18 years and older with a diagnosis of functional dyspepsia attending for evaluation and treatment were recruited. The diagnosis of functional dyspepsia was based on Rome III criteria [1]. Standardized work-up included an upper gastrointestinal (GI) tract endoscopy and laboratory testing. Patients who had erosion or ulceration on endoscopy, a history of GI tract malignancy, liver and biliary tract disease, diabetes mellitus, chronic kidney disease stage IV–V, or psychiatric disorders were excluded. The study was approved by the Medical Research Ethics Committee, Faculty of Medicine Universitas Indonesia and informed consent was obtained from all participants.

### Demographic factors

A separate questionnaire was designed and used to collect demographic characteristics of the participants as covariables. Questions included information on the following variables: age, gender, marital status, education level (primary, secondary, or university), employment status (employed, unemployed) and ethnicity/tribal group. To explore the relationship between demographic variables and HRQoL, we categorized primary and secondary education as low-to-mid education level and university as high education level. Etnicity/tribal categoris were also defined as Javenese or non-Javanese.

### Health-related quality of life

This was assessed using Indonesian version of the Medical Outcomes Study 36-item Short-Form Health Survey (SF-36) [19]. The SF-36 is a widely used and validated measure of generic HRQoL in gastroenterology [8, 20]. It includes one scale for each of eight measured health domains: physical functioning, role-physical, bodily pain, general health, vitality, social functioning, role-emotional, and mental health. These eight domains are aggregated into a physical component summary (PCS) and mental component summary (MCS). All health domain scales are scored using norm-based scores ranging from 0 to 100 with higher scores indicating better health. Norm-based scores can be calculated for each of the health domain scales and a component summary based on 2009 adults US population norms. The population norms for all scale scores and component summaries were 50; thus, scores above and below 50 are above and below the average, respectively, found in the 2009 general population in the USA [21].

### Anxiety and depression disorders

The Hospital Anxiety and Depression Scale was designed to provide a simple, valid and reliable tool for screening of anxiety/depression in medical practice. This consists of seven questions on anxiety and depression on a 4-point (0–3) response scale [22]. This instrument has been translated and has been shown to be valid and reliable for Indonesian patients [23].

### Dyspepsia symptom severity

Symptom severity was assessed by the Short-Form Nepean Dyspepsia Index (NDI), a 10-item questionnaire measuring symptoms and HRQoL in patients with functional dyspepsia. Each item is measured on a 5-point Likert scale ranging from 1 (not at all) to 5 (extremely). A total sum score for each of the five subscales was calculated by adding up the scores for each item (range of total score 10–50, range of each subscale 2–10) with higher scores indicating worse symptom severity [24, 25]. The Indonesian-translated version of the NDI has been validated and shown to be reliable in our population [26].

### Statistical analysis

There were no missing data in any of the questionnaires. Categorical data are described as numbers and percentages [n (%)]. Continuous data are presented as the mean (Standard deviation [SD]) or median (range). The variables investigated were age, gender, symptom severity, employment status, anxiety, depression, and ethnicity. All variables were analyzed univariately to determine associations with the PCS and MCS using parametric or nonparametric tests where appropriate. Two different multiple linear regression models were built to identify any independent variables associated with the PCS and MCS. The variables used in the regression analyses were those with *p* < 0.25 in the univariate analyses. The association was reported using partial r^2^ values. Tolerance values < 0.1 indicated multicollinearity and were used as a criterion to remove the variable from the model. *p* < 0.05 was considered statistically significant. All statistical analyses were performed using the Statistical Package for the Social Science (version 21; IBM Corp., Armonk, NY, USA).

## Results

### Demographic and clinical characteristics

A total of 124 patients with functional dyspepsia with mean age of 47.9 years (SD = 14.4) were evaluated. Eighty-seven patients were female (70.2%) and 37.9% were Javanese. The median NDI score was 22 (range 10–46; Table 1).

**Table 1:** Demographic and clinical characteristics

Age (years)^a^	47.9 (14.4)
Female [*n* (%)]	87 (70.2)
Employment status [*n* (%)]	
Unemployed	79 (63.7)
Employed	45 (36.3)
Education level [*n* (%)]	
Primary and secondary school	70 (56.5)
University degree	54 (43.5)
Ethnicity [*n* (%)]	
Javanese	47 (37,9)
Sundanese	22 (17.7)
Bataknese	17 (13.7)
Others	38 (30.7)
NDI score^b^	22 (10–46)
HADS anxiety score^b^	7 (1–16)
HADS depression score^b^	5 (0–13)

*SD* standard deviation, *NDI* Nepean Dypepsia Index, *HADS* Hospital Anxiety and Depression Scale

^a^ Mean (SD)

^b^ Median (range)

### Health-related quality of life

All domains of the HRQoL except for vitality were impaired in these patients. The domains with the lowest scores were role-emotional, followed by role-physical. The mean PCS score was 42.3 and the mean MCS score was 47.8 (Table 2).

**Table 2:** SF-36 individual domains score

Domain	Scores
Physical functioning^b^	44.2 (23.1–57.5)
Role-physical^b^	41.4 (21.2–57.2)
Bodily pain^b^	42.2 (21.7–62)
General health^a^	44 (9.4)
Vitality^b^	52.6 (31.8–70.4)
Social functioning^b^	42.3 (17.2–57.3)
Role-emotional^b^	38.8 (14.4–56.2)
Mental health^b^	48.3 (19.5–64)
Physical component summary^a^	42.3 (8.4)
Mental component summary^a^	47.8 (10)

*SF-36* short form-36, *SD* standard deviation

^a^ Mean (SD) based on 2009 US population norms [21]

^b^ Median (range) based on 2009 US population norms [21]

### Univariate analysis of associations with HRQoL

Anxiety, female gender, low-to-mid education level, and greater symptom severity were associated with impaired PCS. While impaired MCS was associated with anxiety, depression, and greater symptoms severity. Increased age, ethnicity and employment status were not associated with impaired PCS or MCS (Table 3).

**Table 3:** Univariate analysis between variables analyzed and Short Form-36 physical and mental component summary

Variable	r	t	*p*
Physical component summary			
Gender		−2.57^a^	0.011
Employment status		−1.65	0.083
Education level		−2.35^b^	0.021
Ethnicity		−0.45	0.652
Age	−0.15		0.108
Symptom severity	−0.43		< 0.001
Anxiety	−0.19		0.032
Depression	−0.16		0.075
Mental component summary			
Gender		1.29	0.201
Employment status		−1.08	0.281
Education level		−0.27	0.789
Ethnicity		−0.118	0.906
Age	0.03		0.740
Symptom severity	−0.45		< 0.001
Anxiety	−0.46		< 0.001
Depression	−0.51		< 0.001

^a^ Women had lower mean PCS score (41) than men (45.2)

^b^ Patients with primary and secondary education had lower mean PCS score (40.7) than university degrees (44.3)

### Multiple linear regression of associations with HRQoL

The PCS score was associated with increasing age (*p* = 0.002), female gender (*p* = 0.006), low-to-mid education level (*p* = 0.015), and greater symtom severity (*p* < 0.001). The model explain 36% of the variance in PCS. Female gender (*p* = 0.047), greater symptom severity (*p* = 0.002), anxiety (*p* = 0.001), and depression (*p* = 0.002) were associated with MCS. The model explain 41% of the variance in MCS. Symptom severity made the largest unique contribution (β = 0.52) to the PCS, while anxiety (β = 0.29) was the greatest contributing factor to MCS (Table 4). There was no multicollinearity in the multiple regression model (all tolerances > 0.1).

**Table 4:** Multiple linear regression betwen predictors variables and Short Form-36 physical and mental component summary

Variable	β	Partial r^2^	*p*	R^2^
Physical component summary				
Age	0.26	0.08	0.002	0.36
Gender	0.23	0.06	0.006	
Education level	0.20	0.05	0.015	
Employment status	0.03	0.001	0.701	
Symptom severity	0.52	0.24	< 0.001	
Anxiety	0.01	< 0.001	0.691	
Depression	0.04	0.001	0.913	
Mental component summary				
Gender	0.15	0.03	0.047	0.41
Symptom severity	0.25	0.08	0.002	
Anxiety	0.29	0.09	0.001	
Depression	0.27	0.08	0.002	

## Discussion

Our study confirms previous studies that showed a decrease in the HRQoL in patients with functional dyspepsia [10, 13–15]. We also found that functional dyspepsia mainly affected the physical components rather than mental components of HRQoL. This finding is consistent with two reported studies in Western populations [10, 12].

Several studies have reported bodily pain as one of the most disrupted domains in patients with functional dyspepsia [11, 12, 27]. A recent publication by Shetty et al. using the EuroQol Group EQ-5D questionnaire also demonstrated that 308 of 311 patients with functional dyspepsia complained of impairment in the pain dimension [28]. In our study, the bodily pain scale score was the second lowest among the SF-36 physical component domains. Its scale score was almost whole SD value below the general population norms.

Vitality was the only domain with a scale score above the population norms but role-emotional was the lowest scale score domain. These findings suggest that functional dyspepsia may inhibit our patients from performing their work, but do not cause loss of their life enthusiasm and energy. In contrast to our findings, earlier studies in Western population reported decreased vitality domain value to population norms [11, 12]. Aro et al. also reported vitality as the most affected domain in patients with functional dyspepsia [11].

Impaired HRQoL occurs not only in those functional dyspepsia, but also in other functional GI disorders. Gralnek et al. found HRQoL reduction in patients with irritable bowel syndrome (IBS) [29]. Nevertheless, compared with our study, patients with IBS had a more negative impact on mental components. Decreased HRQoL was also demonstrated in patients with gastroesophageal reflux disease (GERD) [30]. However, the overall HRQoL in patients with GERD was better than the patients with functional dyspepsia in our study.

Female patients with functional dyspepsia generally have lower HRQoL mainly in the physical components compared with male patients [31]. These HRQoL differences are not surprising given data from previous studies reporting that female patients tend to complain of symptoms of dyspepsia than male patients [32]. Here, we found that the HRQoL of female patients was worse than among male patients. Multivariate analysis also demonstrated association between gender and SF-36 PCS and MCS results. However, this latter finding differs from previous studies that showed an association of gender only with PCS [10, 13].

Our study confirms a previous study demonstrated an association between increased age and decreased physical components of HRQoL [10]. The increased prevalence of chronic disease in old age is thought to be closely related to this [33]. Our study did not find any association between employment status and HRQoL. However, this finding needs to be interpreted carefully as most (64%) of the patients in this study were unemployed. In contrast to our research, Oudenhove et al. showed a trend between employment status and PCS [12]. A study in patients with inflammatory bowel disease also demonstrated an association between being unemployed patients with worse HRQoL and more severe anxiety and depression symptoms [34].

In this study, symptom severity was associated with both PCS and MCS. These findings are in line with results from previous studies [10, 13]. Talley et al. also reported different impact on HRQoL for different dyspepsia symptoms [10]. Haag et al. further showed differences in HRQoL between patients with postprandial distress syndrome and those with epigastric pain syndrome [13]. In general, anxiety and depression adversely influence the HRQoL in patients with functional GI disorders [35]. Our study confirms previous research finding association between anxiety and depression and the mental components of HRQoL. However, in contrast to our study, Haag et al. also showed the impact of depression on the physical component of HRQoL [14]. The more significant effect of the psychological factor on HRQoL in that study related to more severe symptoms of anxiety and depression than in our research.

Previous studies in patients with asthma and multiple sclerosis reported an association of education level with HRQoL [36, 37]. Our study also found a similar finding, i.e., a lower level of education was associated with a worse HRQoL in these patients with functional dyspepsia. The differences in HRQoL between low-to-mid and high education level patients might be related to a stronger disease awareness and better coping ability with the challenges of better chronic diseases among patients with higher levels of education [37].

Indonesia comprises a variety of peoples, religions, and languages, with over 600 ethnic groups; the Javanese ethnic group is the largest at 40% of the national population [38]. We believe that sociocultural differences among ethnic groups might have impacts on HRQoL. However, in this study, we found there were no HRQoL differences between Javanese and non-Javanese patients with functional dyspepsia. Nevertheless, most of our patients were living in the same urban area (Jakarta) and not in their area of origin. We hypothesize that living in the same environment will lead to eliminating sociocultural differences between ethnic groups. Our results do not confirm the results of HRQoL studies of coronary heart disease and cancer that showed HRQoL differences among ethnic groups in the United States [39–41]. Those discrepancies were associated with differences in patient perception and knowledge of the disease, medication adherence, and utilization of health facilities between groups [42].

To the best of our knowledge, this is the first study in an Asian population to assess factors associated with HRQoL in patients with functional dyspepsia. We also analyzed factors that have never been studied before, such as education level and ethnicity. We measured HRQoL using the SF-36 norm-based scoring method to enable comparisons with other HRQoL studies.

There were several limitations to this study. One was a potential bias caused by errors in answering the question because we only used self-reporting questionnaires. Another limitation was the cross-sectional design that did not allow us to determine any causal relationships between variables.

Finally, although the decreased HRQoL in patients with functional dyspepsia was clear, further research efforts are still needed in this area. For example, we need to evaluate the potential role of other factors in such patients, such as social supports, household income, financial burdens, religious coping, and comorbidities with other functional gastroduodenal diseases.

## Conclusions

This study showed significant HRQoL impairment in patients with functional dyspepsia in Indonesia. Several essential factors including anxiety, depression, increasing age, female gender, greater symptom severity, and low-to-mid educational level were associated with decreased HRQoL. Our results indicate the importance of HRQoL assessment in such patients. Further, comprehensive management strategies including medical treatments as well as psychosocial support are keys for improving the HRQoL in patients with functional dyspepsia.

## Abbreviations

EPS: Epigastric pain syndrome
GERD: Gastroesophageal reflux disease
HADS: Hospital Anxiety and Depression Scale
HRQOL: Health-related quality of life
IBD: Inflammatory bowel disease
IBS: Irritable bowel syndrome patients
MCS: Mental component summary
NDI: Nepean Dyspepsia Index
PCS: Physical component summary
PDS: Postprandial distress syndrome
SF-36: Short Form-36
